# Blood-based Biomarkers of Alzheimer’s Disease and Neurodegeneration in an Indigenous African Cohort using both SIMOA and NULISA Platforms

**DOI:** 10.21203/rs.3.rs-7688955/v1

**Published:** 2025-10-26

**Authors:** Tolulope Akinyemi, Ilaria Pola, Kubra Tan, Oladotun Olalusi, Joseph Yaria, Gabriel Ogunde, Wiebke Traichel, Nesrine Rahmouni, Olabode Oguntiloye, Ayotomiwa Fagbemi, Eniola Cadmus, Femi Popoola, Joseph Therriault, Mayowa Ogunronbi, Dorcas Olujobi, Olaoluwa Famuyiwa, Joshua Akinyemi, Tharick Pascoal, Mayowa Owolabi, Pedro Rosa-Neto, Chinedu T. Udeh – Momoh, Olusola Ladokun, Roman Romero-Ortuno, Adesola Ogunniyi, Brian Lawlor, Rajesh Kalaria, Rufus Akinyemi

**Affiliations:** Lead City University; University of Gothenburg; University of Gothenburg; University of Ibadan; University College Hospital; University of Ibadan; University of Gothenburg; McGill University; University College Hospital; University College Hospital; University of Ibadan; University of Ibadan; McGill University; University of Ibadan; University of Ibadan; University of Ibadan; University of Ibadan; University of Pittsburgh School of Medicine; University of Ibadan; McGill University; Wake Forest University School of Medicine; Lead City University; Trinity College Dublin; University of Ibadan; Trinity College; University of Ibadan; University of Ibadan, University College Hospital Campus

## Abstract

**Background:**

In low- and middle-income countries, Alzheimer’s disease and related dementias (ADRD) constitute a growing public health burden. Indeed, the lack of awareness and easy screening tools, such as blood-based biomarkers, leaves many patients undiagnosed. In this study, we explored the core biomarkers of AD in an indigenous African cohort (VALIANT) to assess their relevance and potential utility to aid clinical diagnosis.

**Methods:**

Nigerian African older adults (n = 967; ≥50 years) participating in the VALIANT study completed a baseline cross-sectional evaluation with associated clinical diagnosis. We quantified phosphorylated tau (p-tau 217), glial fibrillary acidic protein (GFAP), neurofilament light (NfL), and amyloid beta (Aβ42 and Aβ40) levels in plasma with both the Single Molecule Assay (SIMOA, Quanterix) and Nucleic acid-Linked Immuno-Sandwich Assay (NULISA, Alamar) platforms.

**Results:**

In agreement with previous findings, core AD biomarkers were associated with disease severity both in clinical diagnostic and clinico-pathological groups, with stepwise increases of p-tau 217, NfL and GFAP from cognitively unimpaired (CU) to dementia (*p* < 0.05). These results were consistent across both SIMOA and NULISA platforms. Comparison between sexes showed higher levels of biomarkers in male participants across diagnostic groups. We identified a significant effect of apoE E4 proteotype on p-tau217 levels after adjusting for age and sex but no significant effect on the other AD biomarkers.

**Conclusion:**

This first application of cutting-edge plasma AD biomarker immunoassay using two ultrasensitive platforms in an indigenous African cohort showed good concordance and underscores the relevance and utility of blood-based biomarkers of AD in diverse populations. Additionally, sex differences could unveil biological distinctions inherent in the African population.

## Introduction

The global burden of Alzheimer’s disease and related dementias (ADRD) is growing steadily, with the number of affected individuals projected to increase from the current 55 million to 150 million by 2050^1^. This is particularly critical among ethno-racial groups in low- and middle-income countries (LMICs), including those in Africa, where global AD research has been largely underrepresented mainly due to low awareness, late presentation and limited resources and infrastructure^2, 3^. Such limitations underscore the urgent need for robust studies with equitable representation of African geographic, genetic and cultural diversity.

From a neuropathological perspective, AD is characterized by the deposition of extracellular amyloid-β (Aβ)-containing plaques and the formation of intraneuronal tau tangle aggregates with a pre-symptomatic phase up to 20 years.^4, 5^ Cerebrospinal fluid (CSF) assays for AD biomarkers and amyloid positron emission tomography (PET) are clinically used to assist the diagnosis of AD, but their high cost and invasive nature limit their utility, particularly in LMICs^5^. In this context, blood-based biomarkers offer a promising, non-invasive, scalable and cost-effective alternative, providing a substantial window of opportunity for detection and intervention aimed at slowing disease progression^6, 7^. Indeed, timely identification of Alzheimer’s disease (AD) pathology is critical to enabling early intervention particularly in the prodromal stages of the disease when emerging therapies are most effective^8^.

Blood-based biomarkers provide insights into different aspects of AD pathology. Plasma tau phosphorylated at residue 217 (p-tau217) has demonstrated high accuracy in detecting AD pathology^9–11^, with strong associations with CSF and PET biomarkers of AD^12, 13^, while other biomarkers, particularly neurofilament light protein (NfL), a marker of neurodegeneration, and glial fibrillary acidic protein (GFAP), an astrocytic reactivity marker, provide important complementary information and a more comprehensive assessment of disease pathology^14, 15^.

The utility of these biomarkers has been largely documented among Non - Hispanic White (NHW) populations, but other ethnic groups have been underrepresented. Early efforts involving African populations have been restricted to studies with small sample sizes, limiting their power and generalizability^16–18^. Other studies have reported racial disparities in blood-based neurodegeneration biomarker profiles. For instance, Xiong et al. found that African ancestry individuals enrolled in AD research studies exhibited higher mean plasma Aβ42/40 levels compared with NHW individuals in similar conditions, potentially reflecting lower amyloid pathology in that group^19^. Further studies in cohorts with sociocultural and ethnic diversity are needed to address the current research gap in LMIC populations and to implement the clinical translation of plasma biomarkers for AD in the global population.

This study aimed to determine the plasma concentrations of five biomarkers of AD pathology (amyloidβ −40, amyloidβ–42, p-tau217, GFAP and NfL) among 967 community-dwelling older Nigerian Africans and explore their association with cognitive phenotypes.

## Methods

### Study Site and Setting

Vascular heAlth, fraiLty and cognition in Ageing Nigerians Study (VALIANT) is a longitudinal community-based cohort study aimed at exploring the association between cardiovascular health, cognition, and frailty in Nigeria^20, 21^. A multistage cluster sampling method was employed to recruit 1031 study participants (≥ 50 years) from an urban settlement in Yemetu, Ibadan North Local Government Area, Oyo State, Southwest Nigeria. Participants had been resident in the study area for at least six months and had no evidence of another existing neurologic or psychiatric disorder. Using the African Rigorous Innovative Stroke Epidemiological Surveillances (ARISES) database as a sampling frame, two wards (Ward 3 & 4) were purposively selected^22^. ARISES is an ongoing observational cohort study in selected wards in Ibadan North and Ibarapa Central local government areas in Oyo State, Nigeria. Wards 3 & 4 were purposively selected for the VALIANT study because they host more indigenous urban dwellers than Ward 1, which is predominantly a government reservation area for offices and other establishments. Thereafter, 11 out of the 16 clusters from the two wards were randomly selected and all households within the selected clusters were visited to recruit eligible participants. Ethical approval was obtained from the University of Ibadan/University College Hospital (UI/UCH) Health Research Ethics Committee (HREC) (UI/EC/20/0508) and the research was conducted in accordance with the Declaration of Helsinki.

For comparative analysis, particularly focusing on the effects of APOE, participants from the Translational Biomarkers in Aging and Dementia cohort (TRIAD), a longitudinal imaging and biofluid cohort study of aging and AD, were included. Informed consent was obtained from all participants, and the studies were approved by the relevant ethics boards. Two hundred seventy (270) participants, categorized as cognitively unimpaired (CU), mild cognitive impairment (MCI), AD dementia were included for comparative analysis. Detailed information about this cohort has been previously reported^23^

### Sociodemographic Data

Sociodemographic data were collected from all participants, including sex (male and female), age, level of education (up to primary [< 8 years of studies], secondary [between 8 and 12 years of studies] and beyond secondary [> 12 years of study]), marital status (married and others that includes single, widowed or separated) and Yoruba ethnicity.

### Cardiovascular Health Evaluation (CVH) and evaluation of co-pathologies.

Participants underwent a battery of baseline cardiovascular and other health assessment tools. Components of the CVH metric included blood pressure, fasting glucose, total cholesterol, body mass index (BMI), physical activity, diet, and smoking. Systolic blood pressure and diastolic blood pressure were measured by a mercury sphygmomanometer on the right arm with the subject in a sitting position after 10 miutes of rest. The average of two measurements 5 minutes apart was used in the statistical analyses. Venous blood samples were drawn for the measurement of glucose and lipid profiles after an overnight fast. Fasting lipid profile and fasting plasma glucose were assessed using standard laboratory techniques. BMI was calculated from the height and weight measurements of participants. Physical activity, smoking status, and dietary pattern were assessed using a standardized questionnaire^20^. Furthermore, information about other diseases was also collected. These included sleep disorders, migraine and chronic kidney disease, heart disease including hypertension, stroke, transient ischemic attack (TIA), arrhythmia, atrial fibrillation, haemoglobinopathies, metabolic disease, including hyperlipidemia, diabetes mellitus and obesity, respiratory diseases including chronic bronchitis, bronchitis and asthma/wheezing and infectious/tropical diseases (eg HIV, tuberculosis and malaria).

### Cognitive Assessment

Cognitive function was assessed using translated and validated neurocognitive assessment batteries: Montreal Cognitive Assessment (MoCA), and the Identification and Intervention for Dementia in Elderly Africans (IDEA) cognitive screen^24, 25^. The IDEA and MoCA are tests of general cognitive functioning that have been well-validated in the African setting.^26, 27^ Participants were initially screened for cognitive status using the IDEA (< 9; cognitively impaired vs cognitively unimpaired)^26^ and MoCA (< 19; cognitively impaired/cognitively unimpaired)^27^, and functional impairment was assessed using the IDEA-ADL (< 11)^28^ and FAS scores (> 9)^29^. Cognitive diagnosis [cognitively unimpaired (CU), mild cognitive impairment (MCI) and dementia (Dem)] was made through the process of consensus diagnosis involving two neurologists.

### Blood-based Biomarker Analysis

Biomarker data were available for the study participants. Ten (10) milliliters of whole blood sample were collected at the study site from each participant into EDTA tubes and transported to the laboratory under standard conditions.^30^ The blood samples were centrifuged at 4000rpm for 10mins within 2 hrs of collection and the separated fractions aliquoted into different labelled polypropylene tubes (plasma for biomarker analysis, buffy coat for genomic analysis and red cell concentrates for lipid analysis). These were stored frozen at −20°C for up to 4 weeks and then transferred into − 80°C freezer until shipment for analysis. Plasma samples were shipped frozen on dry ice to and analysed at the Department of Psychiatry and Neurochemistry, University of Gothenburg, Gothenburg, Sweden.

Plasma concentrations of p-tau217, NfL, GFAP, Aβ42 and Aβ40 were determined using Simoa HD-X instrument (Quanterix, Billerica, MA, USA) at the Department of Psychiatry and Neurochemistry, University of Gothenburg. Plasma NfL, GFAP, Aβ42 and Aβ40 were quantified using the commercial Neurology 4-plex E kit (#103670; Quanterix) while plasma p-tau217 was analyzed using ALZpath commercial kit (#104371, Quanterix). Plasma concentrations of p-tau217, NfL, GFAP, Aβ42 and Aβ40 were below the detection limit of the assay in 2.5%, 0.3%, 0.2%, 1.3% and 1.5% of the samples, respectively. These samples were also analysed on the ARGO-HT platform, where the NULISA^™^ CNS-disease assay was performed as described previously^31^. Although the NULISA panel quantifies over 120 protein targets, for this study only p-tau217, NfL, GFAP, Aβ42 and Aβ40 were retained, for comparative purposes. Apolipoprotein E4 (apoE4) and apolipoprotein E (apoE) NPQ values were also included for further analysis as a ratio, to inform on apoE E4 proteotype status.

The plasma collection protocol in the TRIAD cohort followed the procedures previously described^23^.

Plasma samples from TRIAD were analysed at the Department of Psychiatry and Neurochemistry, University of Gothenburg. Plasma Aβ42/40, GFAP and NfL were quantified using the commercial Neurology 4-plex E kit, and plasma p-tau217 was quantified by the ALZpath Simoa assay as described previously.^32^ Performance of the assays has been previously reported^32^. These samples were also analysed on the ARGO-HT platform, where the NULISA^™^ CNS-disease assay was performed as described previously^33^. From this analysis, only apoE E4 and apoE NPQ values were included for comparative analysis as a ratio, to inform on *APOE*-e4 proteotype status. Additionally, *APOE* genotypes was determined for TRIAD participants, with the procedure described previously^34^.

### Statistical analysis

Data were summarized using percentages for categorical data, and mean (standard deviation (SD)), for continuous data. Clinical groups were compared using Chi-square test for categorical variables, and Kruskal–Wallis test for continuous variables. Normality of the variables was examined using histograms and quantile-quantile plots For the Simoa biomarkers, raw fluid biomarker concentrations were not normally distributed therefore log transformation (log_10_) improved the distributions.. NULISA biomarkers concentrations are provided as normalized protein expression (NPQ) values, which are in the log_2_ scale; therefore, no further transformation was needed. Amyloid positivity was defined based on plasma p-tau217 values (Simoa), with a cutoff of 0.42 pg/mL as previously published^32^. The participants were therefore also categorized into clinico-pathological groups of CU−, MCI− and NonAD when the p-tau217 values were below the 0.42 pg/mL, and CU+, MCI + and ADD when the p-tau217 values were equal to or higher than 0.42 pg/mL. Differences in plasma biomarker across pathological groups were assessed using ANCOVA with adjustment for age and sex, followed by Tukey’s corrected *post hoc* pairwise comparisons. When evaluating raw values, ANOVA followed by Tukey’s corrected *post hoc* pairwise comparisons was applied. Correlations between biomarkers were assessed using Pearson tests, while locally weighted scatterplot smoothing (LOESS) analysis was used to explore non-linear trends and sex differences in the proteins. T-test was applied in the boxplot analysis to evaluate significant sex differences within pathological groups.

To investigate the effect of *APOE* ε4 status on biomarker levels, *APOE* ε4 carriership was proxied by proteotype analysis using the NULISA apoE E4/apoE ratio, where the cutoff ≥ 0.5 defined *APOE* ε4 carriership, as previously described^35^. For comparative purposes, TRIAD was included at this stage as an external cohort, with similar data. TRIAD and VALIANT cohorts were age-, sex- and diagnosis-matched using nearest neighbor method (R package ‘*matchit’*). After matching, the subsets used in the analysis included 270 matched pairs from TRIAD and VALIANT, respectivelyThe proportions of *APOE* ε4– carriers and non-carriers within each clinico-pathological group were calculated. Within each cohort, linear models were fitted for GFAP, NfL, Aβ42/40, and p-tau217 as dependent variables, and the effect of APOE-ε4 was tested adjusting for age, sex and diagnosis. For TRIAD, the precision of using plasma-based proteotyping of apoE4/apoE assays for classification of *APOE* ε4 carriership status was compared to gold standard *APOE* ε4 genotyping.

To investigate association between comorbidities and the biomarker levels we fitted linear models adjusted for age and sex. Each disease was coded as a binary variable (presence of pathology or absence of pathology) and individual comorbidities were analysed along with five grouped categories: heart disease, metabolic disease, comorbidities, respiratory conditions, and infectious diseases. To control the false discovery rate, p-values were adjusted using the Benjamini–Hochberg procedure (α = 0.05).

## Results

### Participants and biomarker characteristics.

Overall, 967 participants enrolled in the VALIANT study had available blood-based biomarkers data available from the two assay platforms. The average age of the sample was 65.0 (SD ± 10.4) years, 26.6% were men, the average years of formal education was 5.7 (± 4.8) and 15% had cognitive impairment, as MCI or dementia (Table 1). Plasma p-tau217, GFAP and NfL concentrations were significantly higher in cognitived impaired participants versus CU participants. The demographic and biomarker information for the clinico-pathological groups are presented in **Supplementary Table 1**.

For the comparison analysis, we utilized TRIAD cohort, which included 270 participants; mean age was 70.4 (± 8.8) years, 38.1% were men, individuals had in average 15.4 (± 3.7) years of education and 46% had cognitive impairment.

### Core biomarkers across cognitive status.

**Figure 1.**
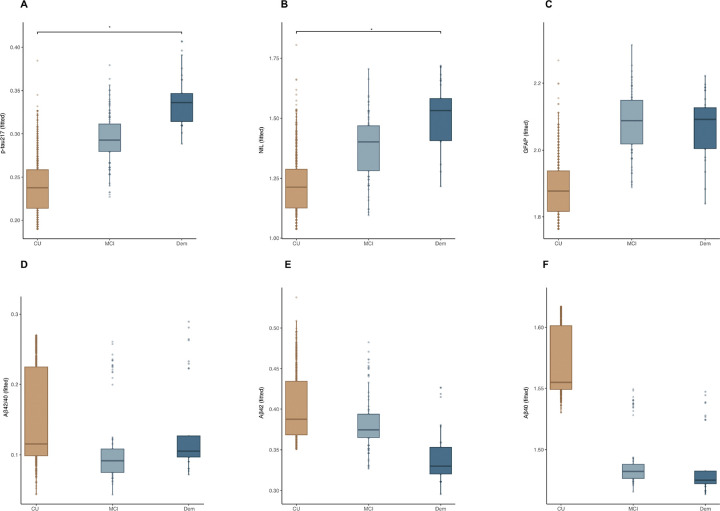
Fitted ANCOVA values (adjusted for age and sex) showing plasma biomarkers differences across the diagnostic groups. Tukey’s corrected post hoc pairwise comparisons were used to determine the significance of biomarker differences between the clinical groups (*p < 0.05, **p < 0.01, ***p < 0.001) **A**. p-tau217. **B.** NfL. **C.** GFAP. **D.** Aβ42/40. **E.** Aβ42. **F.** Aβ40.

When comparing biomarker levels across clinical groups (Fig. 1a–f), levels of plasma p-tau217, NfL and GFAP, showed a stepwise increase. In particular, for both p-tau217 and NfL, we observed significant difference across cognitive status (F(2, 954) = 12.61, *p* < 0.001, partial η^2^ = 0.03 for p-tau217, F (2, 953) = 35.40, *p* < .001, partial η^2^ = 0.07 for NfL), with the difference between CU and dementia remaining significant after Tukey correction for both biomarkers (p < 0.05). Plasma GFAP showed a similar trend of increase across clinical groups (F (2, 954) = 7.01, *p* < .001, partial η^2^ = 0.01), however, no pairwise differences reached significance after Tukey’s correction (*p* > 0.05). As for Aβ42/40 ratio, a tendency to lower levels in patients with MCI and dementia, compared to CU could be appreciated, but no significant diagnosis effect (F (2, 940) = 0.05, *p* = 0.94, partial η^2^ = 0.00). Specifically, across the clinical groups, Aβ40 (F (2, 940) = 2.84, p = 0.05, partial η^2^ = 0.00) showed to decrease from CU to MCI, with progressively lower levels in dementia, while Aβ42 F(2, 944) = 0.62, p = 0.54, partial η^2^ = 0.00) showed a notable decrease from CU to MCI, but remaining relatively stable in the dementia group. The detailed *p* values from Tukey’s post hoc comparisons can be visualized in **Supplementary Table 2.** For transparency, analysis with the raw values can be visualized in **Supplementary Fig. 1.** and **Supplementary Table 3.**

### Core biomarkers in clinico-pathological groups.

**Figure 2.**
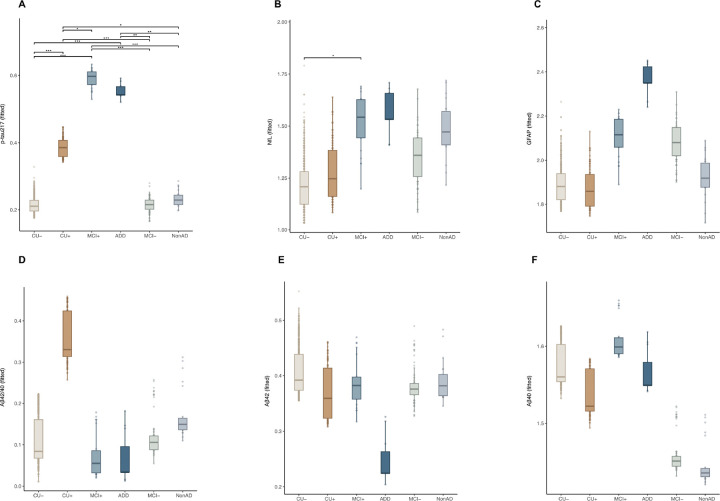
Fitted ANCOVA values (adjusted for age and sex) showing plasma biomarkers differences across the clinico-pathological groups. Tukey’s corrected post hoc pairwise comparisons were used to determine the significance of biomarker differences between the clinico-pathological groups (*p < 0.05, **p < 0.01, ***p < 0.001) **A**. p-tau217. **B.** NfL. **C.** GFAP. **D.** Aβ42/40. **E.** Aβ42. **F.** Aβ40.

When comparing biomarker levels across clinico-pathological groups (Fig. 2a–f), levels of plasma p-tau217, NfL and GFAP, showed higher stepwise increase in the amyloid-positive groups, from CU + to MCI + and to ADD and the same directional pattern in the amyloid-negative groups, though of smaller magnitude. Specifically for p-tau217, we observed a strong diagnosis effect (F[4, 951] = 81.1, *p* < 0.001, partial η^2^ = 0.30), with CU+, MCI+, and ADD showing significantly higher concentrations compared to CU− (all with *p* < 0.05). Similarly, NfL and GFAP showed a strong diagnosis effect (F[4, 950] = 17.12, *p* < 0.001, partial η^2^ = 0.08 for NfL and F[4, 951] = 3.69, *p* < 0.001, partial η^2^ =0.02 for GFAP), with NfL showing elevated levels in MCI + and ADD compared with CU− (*p* < 0.05), while GFAP showing no significant pairwise differences after Tukey’s correction (*p* > 0.05). As for Aβ42/40 ratio, a tendency to lower levels in MCI + and dementia + groups, compared to CU + could be appreciated, but overall, no significant diagnosis effect (F (2, 940) = 1.41, *p* = 0.2, partial η^2^ = 0.008). Notably, the amyloid-negative groups followed the opposite trajectory, with an increase from CU− to MCI− to NonAD dementia. Additionally, Aβ40 (F (2, 940) = 2.10, p = 0.06, partial η^2^ = 0.01) showed to decrease from CU + to MCI+, with progressively lower levels in ADD, while exhibited minimal changes in the amyloid-negative groups. Finally, Aβ42 (F(2, 944) = 0.79, p = 0.55, partial η^2^ = 0.004) showed decreased levels from MCI + to ADD, with similar trends in the amyloid-negative groups. The detailed *p* values from Tukey’s post hoc comparisons can be visualized in **Supplementary Table 4.** For transparency, analysis with the raw values can be visualized in **Supplementary Fig. 2** and **Supplementary Table 5.**

### Correlation of core biomarkers.

**Figure 3.**
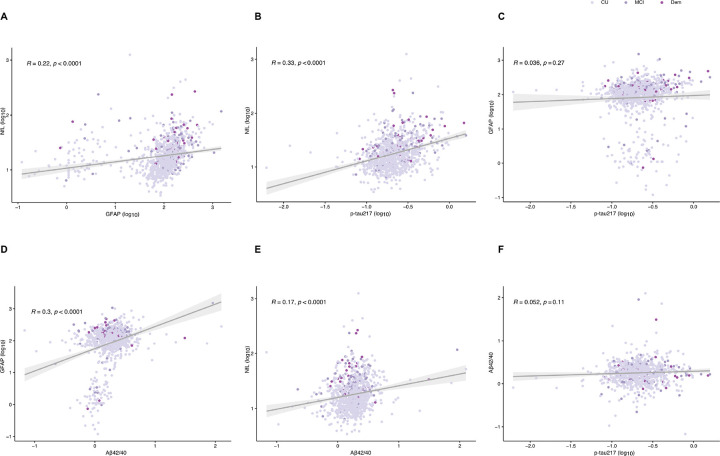
Scatterplot showing the correlation between the plasma biomarkers using Spearman rank correlation. Spearman’s rho and p-values are show on top of each panel. **A**. correlation between NfL and GFAP. **B.**correlation between NfL and p-tau217. **C.** correlation between GFAP and p-tau217. **D.** correlation between GFAP and Aβ42/40. **E.**Correlation between NfL AND Aβ42/40. **F.** correlation between Aβ42/40 and p-tau217.

To understand the relationship between the biomarkers, correlations were performed across all subjects (Fig. 3a–f) and within clinical groups (**Supplementary Fig. 3**). P-tau217 showed robust correlations with NfL (R = 0.33, (CI: 0.27–0.38), *p* < 0.0001) and weaker with GFAP (R = 0.03, (CI: −0.027–0.098, *p* = 0.27), with stronger associations in the MCI (R = 0.47, (CI: 0.31–0.61), *p* < 0.0001 ) and dementia groups (Dementia (R = 0.33, (CI: −0.01–0.60), *p* = 0.06) for Nfl and MCI (R = 0.13, (CI: −0.51–0.32), *p* = 0.15) and Dementia (R = 0.12, (CI: −0.22–0.44), *p* = 0.5) for GFAP. In addition, NfL and GFAP showed robust correlations in the whole sample (R = 0.22, (CI: 0.16–0.28), *p* < 0.0001) but also specifically in the CU (R = 0.23, (CI: 0.16–0.30), *p* < 0.0001) and dementia (R = 0.10, (CI: −0.25–0.43), *p* = 0.5) groups. Lower correlations between Aβ42/Aβ40 and GFAP (R = 0.30, (CI: 0.23–0.35), *p* < 0.0001), Aβ42/Aβ40 and NfL (R = 0.17, (CI: 0.11–0.24), *p* < 0.0001) and Aβ42/Aβ40 and p-tau217 (R = 0.05, (CI: −0.01–0.11), *p* = 0.11) could be observed. In particular, within-group analyses showed that the Aβ42/40 ratio showed a trend of inverse correlation with p-tau217 and NfL only in the dementia group (R= −0.04, (CI: −0.38– −0.38), *p* = 0.8 for p-tau217 and R= −0.001, (CI: −0.35–0.35), *p* = 0.99 for NfL). Finally, Aβ42/Aβ40 showed stronger associations with GFAP in the CU (R = 0.30, (CI: 0.23–0.35), *p* < 0.0001) and MCI groups (R = 0.35, (CI: 0.18–0.51), *p* < 0.001).

### Comparison of core biomarkers between immunoassay platforms.

**Figure 4.**
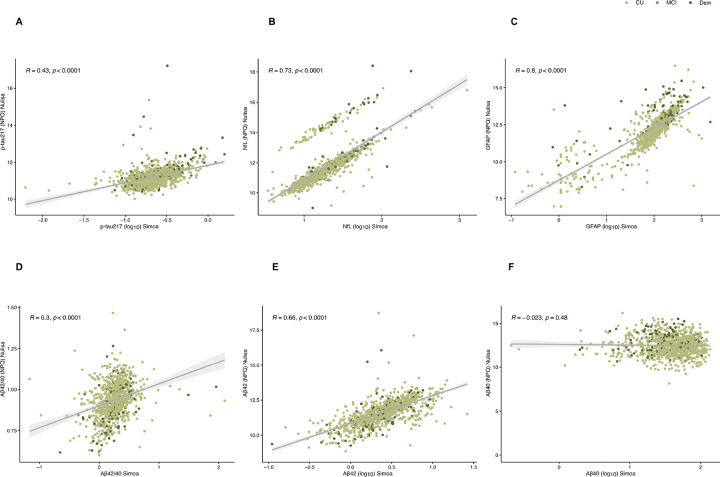
Scatterplot showing correlations between measurements of the same biomarkers on different platforms (Simoa and NULISA) using Spearman rank correlation. Spearman’s rho and p-values are show on top of each panel. **A**. p-tau217. **B.** NfL. **C.** GFAP. **D.** Aβ42/40. **E.** Aβ42. **F.** Aβ40.

When evaluating the correlation between biomarker levels quantified with Simoa and NULISA platforms (Fig. 4a–f), results were convergent, with robust correlations between the measures. Findings showed moderate-to-strong correlations for p-tau217 (R = 0.43, (CI: 0.37–0.47), *p* < 0.0001), NfL (R = 0.73, (CI: 0.69–0.75), *p* < 0.0001), GFAP (R = 0.80, (CI: 0.77–0.82), *p* < 0.0001) and Aβ42 (R = 0.66, (CI: 0.62–0.69), *p* < 0.001). Correlation coefficients were weaker for Aβ42/Aβ40 (R = 0.30, (CI: 0.24–0.36), *p* < 0.001), and not significant for Aβ40 (R=−0.023, (CI: −0.086– −0.040), *p* = 0.48). To further support the similar performance across platforms, we replicated the ANCOVA analysis on the NULISA platform, both for clinical (**Supplementary Fig. 4 and Supplementary Fig. 5)** and clinico-pathological groups (**Supplementary Fig. 6 and Supplementary Fig. 7**). P-tau217, NfL and GFAP showed a stepwise increase from CU to MCI and Dementia. In detail, p-tau217 showed significant difference between CU and dementia (*p* < 0.01) and both NfL and GFAP showed significant difference both between CU and MCI and CU and dementia (**Supplementary Table 6–9).**

### Biomarker changes and sex differences in protein levels

**Figure 5.**
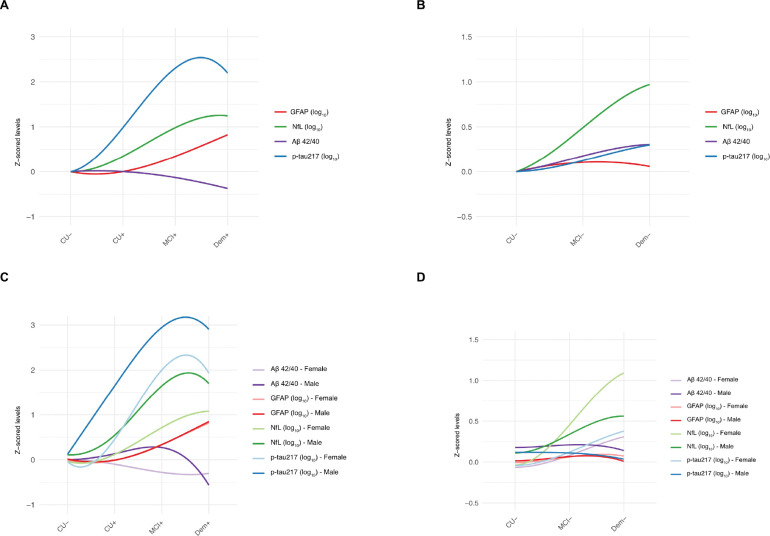
Plasma biomarker trajectories in the cohort using a local weighted regression method (Loess curve). Changes in plasma biomarker levels are represented as Z-scores (anchored to the CU−). Loess represents changes of the biomarkers **A.** in amyloid positive participants, **B.** in amyloid positive participants, stratified by sex, **C.**in amyloid negative participants **D.** in amyloid negative participants, stratified by sex. Lighter colors represent female, while darker colors represent male.

Next, we focused on assessing protein levels throughout a disease pseudo-time, in amyloid-positive and amyloid-negative. In the amyloid positive subset, p-tau217 showed the largest increase across the disease pseudo time from CU + to MCI + to dementia+. GFAP and NfL also showed an increase with disease severity, while Aβ42/40 showed a decreased trend (Fig. 5A). Sex-stratified LOESS trajectories suggested differences in the of biomarker progression. Both in females and males, biomarkers showed similar trajectories for GFAP and p-tau217. At the same time, for NfL, values increased more steeply across the disease continuum in females. In contrast, the, Aβ42/40 showed different trends, with a more stable decrease across disease stages in females, while a steeper decrease from MCI + to dementia + in males. (Fig. 5B) When comparing the LOESS using 95% confidence intervals, male and female trajectories largely overlapped for Aβ42/40 and GFAP across disease groups, suggesting trends but no consistent differences. In contrast, for NfL and p-tau217 the male and female curves diverged at the CU + stages, with non-overlapping confidence intervals. The boxplots of sex differences (**Supplementary Fig. 8)** indicated a trend of difference with higher levels in males than females, with significant differences for Nfl, both in CU+ (p < 0.05) and MCI+ (p < 0.01) and for p-tau217 in CU+ (p < 0.01). In the amyloid negative subset, the magnitude of “change” in biomarker levels was smaller compared with the amyloid positive individuals, with NfL showing the steepest increase (Fig. 5C). Notably, sex-stratified LOESS trajectories suggested differences in the biomarker’s progression. Overall, biomarker levels showed trajectories of steeper increases across the disease continuum in females, whereas in males fewer increases across the stages could be identified. When comparing the LOESS using 95% confidence intervals, male and female trajectories largely overlapped across disease groups, suggesting trends but no consistent differences. (Fig. 5D). When assessing the differences with the boxplots (**Supplementary Fig. 9)**, biomarker levels were slightly higher in males than females, with significant differences between males and femalesfor Aβ42/40, both in the CU− and MCI − groups (both p < 0.05). On the contrary, GFAP showed a trend of higher values in females than males.

### ApoE proteotype associations with biomarker levels.

**Figure 6.**
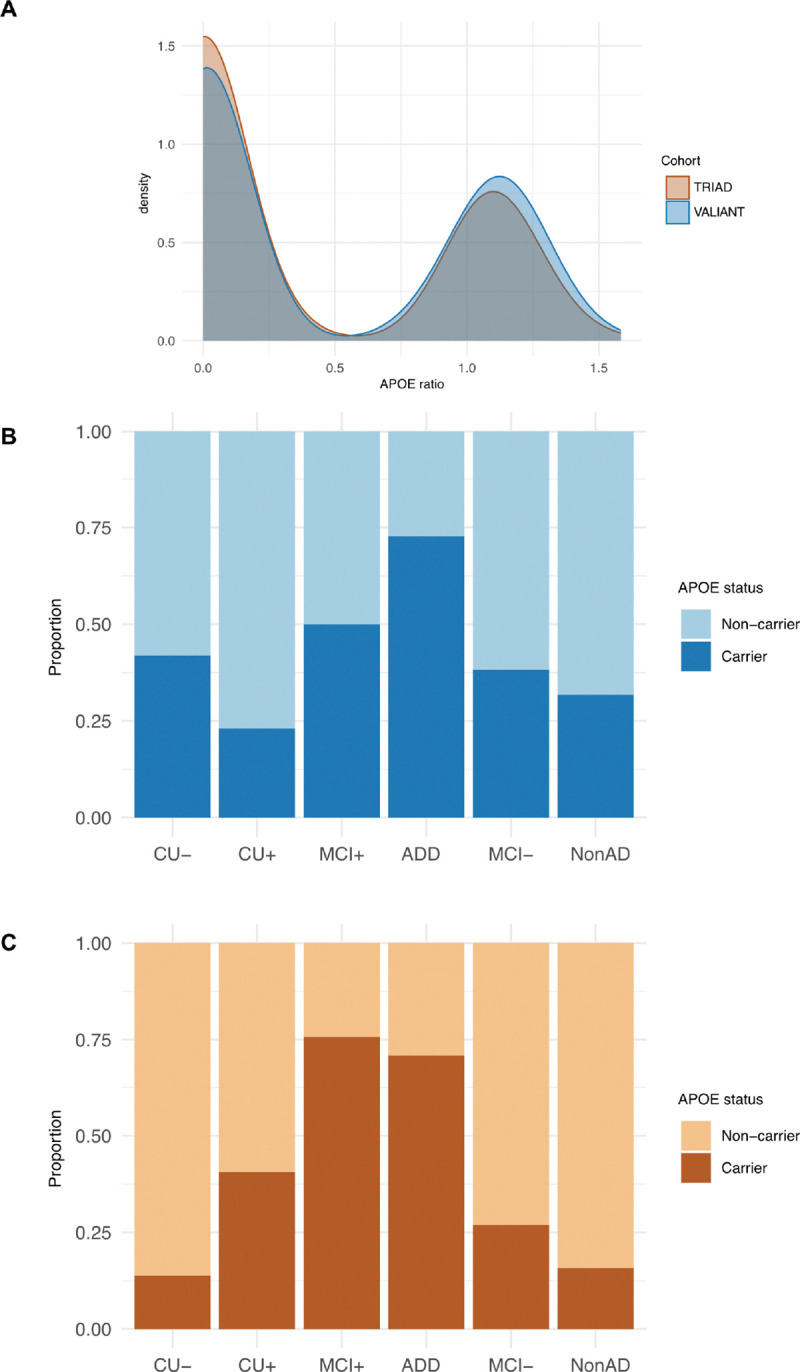
**A.** Density plots showing the distribution of APOE ratio by cohort (VALIANT in blue and TRIAD in orange) and CSF p-tau181/Aβ42. **B. Proportion of *APOE* ε4 carriers by clinico-pathological group for VALIANT cohort. Light blue indicated “non-carrier” status, while dark blue indicates “carrier” status. C. Proportion of *APOE* ε4 carriers by clinico-pathological group for TRIAD cohort. Light orange indicated “non-carrier” status, while dark orange indicates “carrier” status.**

A proteotype analysis proxied *APOE* ε4 carriership status, which was then used to evaluate its association with biomarker levels. For comparative purposes, TRIAD data was also included at this stage and cohorts were age-, sex- and diagnosis-matched. Distribution of APOE ratio proportion of *APOE* ε4 carriers by clinico-pathological group was assessed in each cohort (Figs. 6a–c). In the VALIANT cohort, apoE E4 positivity was significantly associated with higher p-tau217 concentrations (β = 0.07, *p* < 0.05), while no significant associations were observed with GFAP, NfL, or Aβ42/40 ratio. In the TRIAD cohort, apoE E4 positivity was significantly associated with higher pTau217 concentrations (β = 0.22, *p* < 0.001), with a trend of significance also for GFAP (β = 0.13, *p* = 0.055). No significant associations were observed with Aβ42/40 ratio and NfL (**Supplementary Table 10)**. To support the use of the proteotype as a proxy for *APOE*-ε4 carriership status, we examined the agreement between proteotype and genotype data in TRIAD cohort. The apoE4/apoE ratio presented a very well separated distribution between participants stratified according to *APOE* ε4 status classified with genotype analysis. Results are included in **Supplementary Fig. 10**.

### Effects of vascular factors and comorbidities on the biomarker levels.

Finally, it was important to investigate whether the presence of comorbidities would influence biomarker levels. Notably, the individuals who have had past infectious/tropical diseases had lower GFAP, with Malaria driving this association. No other associations were statistically significant, with or without multiple-testing correction and when adjusting for age and sex (**Supplementary Table 11)**.

## Discussion

This study evaluated the potential of plasma biomarkers of AD (p-tau217, GFAP, NfL and Aβ42/40) in a population-based African cohort of older adults (≥ 50 years), by investigating the association of the biomarkers with cognitive status. We reported that core biomarkers changed with disease severity both in clinical diagnosis and clinico-pathological groups, with stepwise increases of p-tau217, NfL and GFAP, and a decrease of plasma Aβ42/40, from CU to dementia. This trend was confirmed with measurements from two different platforms. Furthermore, the biomarkers showed different trends in females and males throughout the disease stages, with male individuals exhibiting more elevated biomarker levels. Lastly, the apoE E4 proteotype demonstrated a significant effect on p-tau217 levels, but no significant effect on the other AD biomarkers. Taken together, our findings highlight the relevance and utility of blood biomarkers as a diagnostic tool in diverse populations including indigenous Africans.

Recent research has unmasked the importance and value of plasma biomarkers as tools for AD evaluation, especially in the LMIC context, where the lack of infrastructure and limited resources impede the use of PET imaging or CSF biomarker assays. From our analysis, the increase of p-tau217, NfL and GFAP in the MCI and dementia groups, compared to the CU group, aligns with current biomarker evidence. Indeed, plasma p-tau 217 has been recognized as a robust AD-specific biomarker^36^ showing a large fold-change in Aβ-positive patients with cognitive impairment ^37^. As shown from our results, as well as in previous research, its level starts to increase in Aβ-positive individuals and keeps increasing throughout the continuum, thus proposing it as a biomarker possibly driven by both Aβ and tau pathologies ^38^. NfL has been proposed as a neurodegeneration biomarker, with higher levels predicting brain atrophy and faster cognitive decline^39^. Indeed, our study revealed higher levels of NfL already in MCI and a stepwise increase to dementia, especially in the amyloid-positive groups. GFAP, an astrocytic activation biomarker, had been previously reported to be elevated in AD and has been introduced as a biomarker of inflammation in the latest criteria from the Alzheimer’s Association Workgroup^36^. In our results, this marker showed high elevation already in the MCI group and showed to be more AD specific and not as elevated in non-AD pathologies. This highlights that GFAP could be a marker for Aβ pathology in line with previous findings^14^. These findings, reinforce the concept that altered p-tau217 levels, together with NfL and GFAP, represent essential biomarkers for dementia and AD pathology, increasing early in the disease process and preceding the onset of cognitive symptoms. Concerning plasma Aβ42/40 ratio, while Aβ42 is an indicator of amyloid plaque accumulation and its levels tend to decrease as the disease progresses, Aβ40 levels tend to remain relatively stable^40^. Our results showed that both Aβ42 and Aβ40 decrease from CU to MCI to dementia, with a surprisingly large drop in Aβ40 contributing to the change. Previous results in a Black cohort showed differences in Aβ42/40 ratio in the Black individuals compared to white as the Black participants had lower baseline Aβ40^19^. Our results suggest that differences in Aβ40 in the African population could potentially bias the interpretation of the ratio across racial groups. Additionally, it is important to highlight that although some studies indicate that pre-analytical conditions can influence biomarker stability of plasma Aβ40 and plasma Aβ42, reporting significant decline after multiple freeze-thaw cycles^41, 42^ antother recent study has shown the relative stability of blood-based AD biomarkers, particularly amyloid and tau, at temperatures of −20 degrees for up to 15 weeks^43^.

Further, we evaluated the differences in biomarker levels between male and female individuals. Indeed, differences in biomarker levels and trends, could be observed, with males showing higher levels of biomarkers compared to females. Existing research has shown sex-related differences in AD, starting with different cognitive psychiatric symptoms, and females usually showing a faster decline after diagnosis^44^. In terms of fluid biomarkers, previous research has shown that women have higher plasma GFAP in CU participants ^14, 45^, while plasma p-tau217 and NfL did not show differences in terms of sex at all disease stages^46^. Controversial results have been reported for Aβ42/40 ratio^47^, with evidence showing no sex differences, while other evidence reported lower Aβ42/40 ratios in females compared to males only in CU participants^48^. From our results, levels of biomarkers were higher in male, especially for NfL and Aβ42/40 ratio, while no differences could be noted for GFAP. This result might be due to males and females having different metabolic, cerebrovascular and socio-economic risk factors^44^, all factors which are particularly important to consider in the African context. Additionally, these differences could unveil biological distinctions inherent to the African population.

Next, we evaluated the effect of *APOE* on the plasma biomarkers. Previous research has shown that the influence of *APOE* ε4 on AD is attenuated in African populations, compared to other. This difference lies in the differential ancestral background surrounding the *APOE* locus^49^. In our study, we made a comparison between a NHW ancestry population cohort (TRIAD) and a native African ancestry population cohort (VALIANT) and we found similar results between the two, with *APOE* status showing an effect on plasma p-tau217. It is important to consider how the two cohorts differ substantially in terms of both demographic and clinical characteristics and therefore precluding an unbiased comparison of *APOE* ε2/ε3/ε4 carrier prevalence across the two cohorts. However, when adjusting for these differences and comparing matched groups, the proteotype data suggest a similar genotype effect across cohorts.

Finally, we evaluated the concordance between different methods for measurement of AD plasma biomarkers, Simoa and NULISA. Our results showed excellent concordance between the two platforms, except for Aβ42/40 ratio. As previously discussed, this would most likely be due to the different freeze-thaw cycles between the two measurements. When replicating the Simoa analysis, with the NULISA data, similar findings could be observed, providing strong support for the results.

This study presents some limitations. First, this cohort lacked PET or CSF data for confirmation of amyloid status, due to limited resources and infrastructures. We also did not clinically subtype the dementia subtypes. To mitigate this issue, we applied a previously validated cut-off to determine amyloid pathology^32^. Second, the cohort showed predominance of CU individuals, rather than MCI and dementia, and females rather than males. This reflected the study’s design, which relies on a community-based cohort, where participants were met in their homes. Women stayed more at home in the African cohort and showed willingness to participate in the study than the males. Third, the study lacked *APOE* genotyping data; however, based on previous reports^35^, the proteotype from NULISA suggests to reliably reflect the genotype. Lastly, the participants of the study presented with significant comorbidity burden; to mitigate this limitation, we assessed the effect of comorbidities on plasma biomarker levels.

Nevertheless, it should be noted that this is a unique pioneering work in Africa with the current largest sample size in any AD biomarker work, with blood samples well-stored and analyzed on two reliable platforms. Additionally, the nature of the study allows wider diversity to ensure optimized implementation, and accounting for higher rates of co-morbidities.

In conclusion, our study provides information about the interpretation of plasma biomarkers for AD in an indigenous African cohort. Our study provides evidence that blood-based biomarkers of AD can be important biomarkers in diverse populations. The current study contributes to the ground work of processes of local validation and adoption of AD and dementia blood-based biomarkers among indigenous Africas.

## Supplementary Material

Supplementary Files

This is a list of supplementary files associated with this preprint. Click to download.

• Supplementarymaterials.docx

## Figures and Tables

**Table 1:** Demographics of the participants by clinical group

	CU (N = 828)^1^	MCI (N = 106) ^1^	Dem (N = 33) ^1^	p-value
**Sex**				<0.05
Male	232 (28.0%)	17 (16.0%)	8 (24.2%)	
Female	593 (71.6%)	89 (84.0%)	25 (75.8%)	
**Age**	64 (± 9.7)	73 (±9.7)	77 (±11)	<0.001
**Years of education**	6.2 (±4.7)	2.5 (±3.7)	1.8 (±3.7)	<0.001
**p-tau217 pg/mL**	0.24 (±0.13)	0.29 (±0.23)	0.34 (± 0.27)	<0.05
**GFAP pg/mL**	120 (±95)	180 (±180)	170 (±110)	<0.001
**NfL pg/mL**	23 (±51)	31 (±29)	47 (± 57)	<0.001
**Aβ40 pg/mL**	47 (± 28)	39 (± 24)	40 (± 24)	<0.05
**Aβ42 pg/mL**	3.2 (±2.5)	3.0 (±1.8)	2.6 (±1.7)	
**Aβ42/40 ratio**	3.2 (±2.5)	3.0 (±1.8)	2.6 (±1.7)	
**Aβ status**				<0.05
Negative	708 (85.5%)	84 (79.2%)	22 (66.7%)	
Positive	120 (14.5%)	22 (20.8%)	11 (33.3%)	

1 N (%); Mean (SD)

2 Pearson’s Chi squared test; Kruskal-Wallis rank sum test
